# Identifying clinically meaningful subgroups following open reduction and internal fixation for proximal humerus fractures: a risk stratification analysis for mortality and 30-day complications using machine learning

**DOI:** 10.1016/j.jseint.2024.04.015

**Published:** 2024-05-06

**Authors:** Avinesh Agarwalla, Yining Lu, Anna K. Reinholz, Erick M. Marigi, Joseph N. Liu, Joaquin Sanchez-Sotelo

**Affiliations:** a Department of Orthopedic Surgery, Westchester Medical Center, Valhalla, NY, USA; b Department of Orthopedic Surgery, Mayo Clinic, Rochester, MN, USA; c Department of Orthopedic Surgery, Baylor Scott & White Medical Center, Temple, TX, USA; d USC Epstein Family Center for Sports Medicine, Keck Medicine for USC, Los Angeles, CA, USA

**Keywords:** Machine learning, Proximal humerus fracture, Open reduction internal fixation, Risk stratification, Risk factors, Complications, Readmission, Reoperation

## Abstract

**Background:**

Identification of prognostic variables for poor outcomes following open reduction internal fixation (ORIF) of displaced proximal humerus fractures have been limited to singular, linear factors and subjective clinical intuition. Machine learning (ML) has the capability to objectively segregate patients based on various outcome metrics and reports the connectivity of variables resulting in the optimal outcome. Therefore, the purpose of this study was to (1) use unsupervised ML to stratify patients to high-risk and low-risk clusters based on postoperative events, (2) compare the ML clusters to the American Society of Anesthesiologists (ASA) classification for assessment of risk, and (3) determine the variables that were associated with high-risk patients after proximal humerus ORIF.

**Methods:**

The American College of Surgeons–National Surgical Quality Improvement Program database was retrospectively queried for patients undergoing ORIF for proximal humerus fractures between 2005 and 2018. Four unsupervised ML clustering algorithms were evaluated to partition subjects into “high-risk” and “low-risk” subgroups based on combinations of observed outcomes. Demographic, clinical, and treatment variables were compared between these groups using descriptive statistics. A supervised ML algorithm was generated to identify patients who were likely to be “high risk” and were compared to ASA classification. A game-theory–based explanation algorithm was used to illustrate predictors of “high-risk” status.

**Results:**

Overall, 4670 patients were included, of which 202 were partitioned into the “high-risk” cluster, while the remaining (4468 patients) were partitioned into the “low-risk” cluster. Patients in the “high-risk” cluster demonstrated significantly increased rates of the following complications: 30-day mortality, 30-day readmission rates, 30-day reoperation rates, nonroutine discharge rates, length of stay, and rates of all surgical and medical complications assessed with the exception of urinary tract infection (*P* < .001). The best performing supervised machine learning algorithm for preoperatively identifying “high-risk” patients was the extreme-gradient boost (XGBoost), which achieved an area under the receiver operating characteristics curve of 76.8%, while ASA classification had an area under the receiver operating characteristics curve of 61.7%. Shapley values identified the following predictors of “high-risk” status: greater body mass index, increasing age, ASA class 3, increased operative time, male gender, diabetes, and smoking history.

**Conclusion:**

Unsupervised ML identified that “high-risk” patients have a higher risk of complications (8.9%) than “low-risk” groups (0.4%) with respect to 30-day complication rate. A supervised ML model selected greater body mass index, increasing age, ASA class 3, increased operative time, male gender, diabetes, and smoking history to effectively predict “high-risk” patients.

Proximal humerus fractures are a common orthopedic injury, accounting for 4%-5% of all fractures.^6^ Traditionally, these fractures have a bimodal distribution, with acute high-energy injuries in a younger population and low-energy injuries in an older population with poor bone quality.^61^ While many proximal humerus fractures are nondisplaced or minimally displaced and can be managed successfully with nonoperative management, the surgical treatment of displaced proximal humerus fractures is more complex. Percutaneous fixation, open reduction and internal fixation (ORIF), and arthroplasty are all viable options; however, treatment selection is complex and varies by surgeon preference.^27^^,^^61^

ORIF is commonly performed for displaced proximal humerus fractures. However, complications such as hardware articular penetration, malunion, nonunion, avascular necrosis, or infection are problematic and not uncommonly observed after ORIF. Delineating prognostic factors for complications after ORIF is of utmost importance and historically has relied on stepwise regression analyses.^36^ Individual factors, including complex fracture pattern, higher American Society of Anesthesiologists (ASA) physical status classification, poor social independence, and underlying comorbidities have known negative associations with surgical outcomes.^13^^,^^37^ These linear factors, in addition to clinical experience, are currently used to help guide surgeon decision-making and predict the possibility of complications. There are, however, important limitations when trying to predict outcomes following ORIF of proximal humerus fractures based on currently available literature.

Patient-reported outcome measures or postoperative complications are highly variable when accounting for baseline patient demographics and comorbidities. Due to the evolving healthcare landscape of bundled payment models, predictive tools are imperative in assisting physicians and healthcare systems in identifying patients who are at risk for complications. Machine learning (ML) has shown some promise in bridging these gaps within the orthopedic literature.^30^^,^^41^^,^^46^ ML clustering analysis is a method used to group patients into categories based on outcomes. Leveraging this technique can result in grouping patients into those with ideal outcomes or “high achievers” and those with suboptimal outcomes or “low achievers” and completely avoid the influence of traditional statistical bias. ML clustering analysis is performed by mathematically minimizing the distance between patient factors while maximizing the distance between groups, thereby objectively stratifying patients. The aims of this study were to (1) use unsupervised ML to stratify patients into “high-risk” and “low-risk” clusters, (2) assess if “high-risk” patients had a greater risk of 30-day postoperative complications, (3) compare the output of ML to ASA classification, and (4) determine which variables were associated with high-risk patients. We hypothesized that our ML model will reliably and effectively predict complications in a subset of patients from the National Surgical Quality Improvement Program (NSQIP) database after proximal humerus ORIF.

## Methods

### Data source

This retrospective cohort study was considered exempt from our Institutional Review Board given the utilization of an anonymized prospectively collected database from the American College of Surgeons (ACS) NSQIP database. We queried this NSQIP database to identify patients who had undergone ORIF of a proximal humerus fractures between 2007 and 2018 using the Current Procedural Terminology codes 23615 (open treatment of proximal humeral [surgical or anatomical neck] fracture, with or without internal or external fixation, with or without repair of tuberosity[s]) and 23630 (open treatment of greater humeral tuberosity fracture, includes internal fixation). All proximal humerus fracture types were included and those with greater tuberosity fractures were also included given the precedent set by Nicholson et al.^15^ Patients aged more than 18 years with any comorbidities were included in the analysis. Polytrauma patients and those with open fractures were excluded. The NSQIP database includes documentation of more than 300 collected variables including patient demographics, comorbidities, intraoperative factors, and perioperative outcomes and complications within the 30-day period following discharge. Our primary outcome of interest was 30-day mortality, while secondary outcomes included 30-day readmission and reoperation rates, as well as perioperative complications.

Variables with less than 35% missing data were imputed using the Miss Forest multiple imputation method according to Rubin’s rule, the threshold of 35% being determined empirically.^53^ Compared to imputation, complete case analysis predisposes to the introduction of biases that can significantly reduce the power of the analysis, and multiple imputations is a commonly used technique for mitigating these risks in large datasets.^25^^,^^26^

### Unsupervised clustering

Following imputation of missing variables, patients were partitioned into clinically distinct subgroups using unsupervised clustering. Clustering is an ML technique that produces optimized groupings of objects based on a specified distance measure within a multidimensional feature space.^38^^,^^60^ User input into the model in this instance is minimal, without supplied features and a specified outcome (hence “unsupervised”). Distinct from supervised ML, this technique is often used for exploratory analysis, dimensionality reduction, and outlier removal, especially when there are multiple outcomes of interest in the data, such as in the case of risk stratification.

Four candidate-unsupervised ML algorithms were selected to partition the patient cohort: Unweighted Pair Group Method with Arithmetic Mean, K-means clustering, agglomerative nesting of hierarchical clustering, and divisive analysis of hierarchical clustering.^10^^,^^11^ The optimal clusters were determined based on minimization of Euclidean distance between patients. Algorithm performance was then evaluated through internal validation metrics, which assess the quality of clustering based on the partitions produced and the subjects within each cluster, and internal stability, which measures the consistency of the results through repeated clustering following an iterative feature-elimination process. We used the internal validation metrics of connectivity and silhouette coefficient, along with the internal stability metrics of average distance (AD) and figure of merit (FOM) to determine the best candidate clustering algorithm.^10^

Briefly, connectivity is a measure of the degree to which nearest neighbors in the feature space are clustered together; it can take a value between 0 and ∞ and should be minimized.^10^^,^^43^ The silhouette coefficient is a proportion of the distance between objects in a cluster to the distance between neighboring clusters and should be maximized. AD measures the changes in AD between observations within the same cluster and FOM measures the variance within each cluster, respectively, following iterative elimination of features. Both of these should be minimized.^10^^,^^42^ The final cluster assignments of patients in the study cohort were visualized using a 3-dimensional principal component analysis, which is a method of dimensionality reduction that mathematically decomposed the collection of patient characteristics (demographics, comorbidities, and preoperative laboratory values) into representative components, enabling them to be plotted on a Euclidean coordinate system.^44^

### Supervised machine learning

After the optimal clusters were formed among the patient cohorts, we developed a supervised ML model to predict the likelihood that a future patient will fall within the high-risk cluster.^33^^,^^45^ Patients were stratified into “high-risk” and “low-risk” groups based upon a 3-dimensional component analysis that included comorbidities, complications, and mortality. These components were clustered and plotted on a 3-dimensional space to observe the degree of separation between the clusters. Supervised learning models were constructed adherent to The Transparent Reporting of a multivariable prediction model for Individual Prognosis or Diagnosis guidelines and the Guidelines for Developing and Reporting Machine Learning Models in Biomedical Research.^14^^,^^35^

Model hyperparameters were tuned using 10-fold cross-validation and validated via 0.632 bootstrapping with 1000 resampled datasets. Bootstrapping has been shown to improve both model variance and bias when compared to internal validation by train-test split. Two models were trained: (1) a stepwise logistic regression model to establish a baseline and (2) an extreme gradient boosting (XGBoost, Seattle, WA, USA) model. The logistic regression model is fitted with the same inputs and serves as a traditional statistics benchmark against which the ML methods are evaluated. The optimal model was chosen based on area under the receiver operating characteristics curve (AUROC), which measures the ability to discern high vs. low risk by the model. Based on the works of Hosmer and Lemeshow, an AUROC of 0.7-0.8 is considered acceptable, 0.8-0.9 is considered good, and > 0.9 is considered excellent.^24^ An additional performance measure assessed was calibration of the model’s predicted probabilities as a function of observed frequencies within the test population is summarized in a calibration plot. An ideal model is a straight line with intercept 0 and slope of 1. Finally, the mean squared difference between predicted probabilities of models and observed outcomes, known as the Brier score, was assessed for each candidate model, with smaller values considered more optimal. The candidate algorithm Brier scores are then compared to the Brier score of the null model, which assigns a predicted probability equal to the outcome prevalence in the study population.

Global Shapley values, which are frequently used as interpretability enhancements for supervised predictive models, were calculated and plotted to illustrate the contributions of input to “high-risk” cluster membership. Shapley values represent a solution to a game-theory problem where the input features are players in a game, and the prediction is the outcome; the final Shapley values were plotted the average marginal contribution of a feature value across all possible coalitions. ML models were compared to ASA score to assess the ability of these models to predict complications in comparison to traditional indices. “Low risk” in ASA was classified as a score of 0, 1, or 2, while “high-risk” patients were identified with scores of 3 or 4.^18^^,^^28^ These groups were used to predict postoperative complications.

### Statistical analysis

All modeling and statistical analysis was performed through the R Language for Programming in RStudio software version 1.1.143 (R Foundation for Statistical Computing, Vienna, Austria). Univariate comparisons between high-risk and low-risk clusters were performed using Welch’s *t*-tests for continuous variables and chi-square analyses for categorical variables. All statistical tests were 2-tailed, and the statistical significance was established with an alpha less than 0.05.

## Results

### Population demographics

A total of 4806 ORIFs for proximal humerus fractures were collected in the NSQIP database during the period of interest. On internal validation of the best performing clustering algorithm and optimal number of clusters, 2 clusters generated via K-means clustering were selected on the basis of internal validation measures (connectivity = 43.5, silhouette = 0.72, AD: 88.8, FOM: 2.11), which indicate reproducible partitioning of the cohort into clusters with the least intragroup differences and the greatest intergroup differences (Supplementary Appendix S1). After clustering and outlier removal, a total of 4670 patients were included in the final cluster-based analysis. Subsequent risk stratification identified 202 patients in the “high-risk” cluster and the rest in the “low-risk” cluster. Comparison of the 2 clusters at baseline demonstrated significant differences in male sex (*P* < .006), race (*P* < .007), ASA classification (*P* < .001), total operation time (*P* = .004), anesthesia type (*P* < .001), dependent functional status (*P* < .001), and wound vac status (*P* < .001) (Table I). Compared with patients in the low-risk cluster, a significantly greater proportion of patients in the high-risk cluster had diabetes, hypertension treated pharmacologically, acute and chronic kidney disease, chronic obstructive pulmonary disease, coagulopathies, and significant dyspnea with exertion, among other comorbidities (Table II).

**Table I:** Demographic and clinical characteristics among patients undergoing open reduction internal fixation stratified by risk.

Variable	Low risk (N = 4468)	High risk (N = 202)	*P*
Age, yr	62.1 (14.2)	65.1 (13.1)	**.003**
Male sex, n (%)	1247 (27.9%)	75 (37.1%)	**.006**
BMI (kg/m^2^)	29.0 (7.5)	29.8 (9.1)	.150
Race			**.007**
White	3476 (77.8%)	155 (76.7%)	
Unknown/not reported	726 (16.2%)	28 (13.9%)	
Black	149 (3.3%)	12 (5.9%)	
Asian	91 (2.0%)	3 (1.5%)	
American Indian/Alaska Native	21 (0.5%)	2 (1.0%)	
Native Hawaiian or Pacific Islander	5 (0.1%)	2 (1.0%)	
ASA classification			**<.001**
No disturbance	300 (6.7%)	2 (1.0%)	
Mild disturbance	2006 (44.9%)	55 (27.2%)	
Severe disturbance	1977 (44.2%)	112 (55.4%)	
Life threatening	185 (4.1%)	33 (16.3%)	
Moribund	0 (0.0%)	0 (0.0%)	
Total operation time in min	110.4 (54.6)	121.6 (63.2)	**.004**
Anesthesia			**<.001**
General	4332 (97.0%)	191 (94.6%)	
MAC/IV	53 (1.2%)	5 (2.5%)	
Other	2 (0.0%)	2 (1.0%)	
Regional	61 (1.4%)	4 (2.0%)	
Spinal	20 (0.4%)	0 (0.0%)	
Dependent functional status	240 (5.4%)	32 (15.8%)	**<.001**
Concomitant Biceps tenodesis	222 (5.0%)	9 (4.5%)	.870
Concomitant Rotator cuff repair	185 (4.1%)	5 (2.5%)	.322
Bone grafting	38 (0.9%)	0 (0.0%)	.360
Tenotomy	31 (0.7%)	3 (1.5%)	.384
Wound vac	0 (0.0%)	4 (2.0%)	**<.001**
Arthroscopic-assistance	57 (1.3%)	0 (0.0%)	.198

*BMI*, body mass index; *ASA*, American Society of Anesthesiologists; *MAC*, monitored anesthesia Care; *IV*, intravenous.

Continuous variables reported as mean (SD), while categorical variables reported as counts (%).

Bold values represent statistical significance (*P* < .05).

**Table II:** Baseline comorbidities among patients undergoing open reduction internal fixation stratified by risk.

Variable	Low risk (N = 4468)	High risk (N = 202)	*P*
DM requiring oral agents or insulin	835 (18.7%)	57 (28.2%)	**.001**
Hypertension requiring medication	2203 (49.3%)	116 (57.4%)	**.029**
Current smoker within 1 yr	957 (21.4%)	51 (25.2%)	.228
Acute renal failure (preop)	0 (0.0%)	11 (5.4%)	**<.001**
CKD	0 (0.0%)	11 (5.4%)	**<.001**
Currently on dialysis (preop)	0 (0.0%)	20 (9.9%)	**<.001**
CHF within 30 days prior to surgery	31 (0.7%)	9 (4.5%)	**<.001**
Dyspnea			**<.001**
At Rest	23 (0.5%)	5 (2.5%)	
Moderate exertion	173 (3.9%)	13 (6.4%)	
No	4272 (95.6%)	184 (91.1%)	
Ventilation	0 (0.0%)	3 (1.5%)	**<.001**
History of severe COPD	255 (5.7%)	23 (11.4%)	**.001**
Ascites within 30 days prior to surgery	0 (0.0%)	4 (2.0%)	**<.001**
Disseminated cancer	51 (1.1%)	9 (4.5%)	**<.001**
History of open wound with or without infection	75 (1.7%)	10 (5.0%)	**.002**
Steroid use for chronic condition	145 (3.2%)	11 (5.4%)	.133
>10% loss of body weight in last 6 months	0 (0.0%)	18 (8.9%)	**<.001**
Bleeding disorders	178 (4.0%)	17 (8.4%)	**.004**
Transfusion of ≥ 1-unit RBCs in 72 hours prior to surgery	62 (1.4%)	13 (6.4%)	**<.001**

*DM*, diabetes mellitus; *CKD*, chronic kidney disease; *CHF*, congestive heart failure; *COPD*, chronic obstructive pulmonary disease; *RBC*, red blood cell.

Categorical variables reported as counts (%).

Bold values represent statistical significance (*P* < .05).

Comparative analysis between the 2 clusters demonstrated that patients in the “high-risk” cluster had a significantly increased rate of 30-day mortality following ORIF compared to the “low-risk” cluster (0.4% vs. 8.9%, *P* < .001). Additionally, the “high-risk” cluster demonstrated significant differences in the following secondary outcomes: 30-day readmission rates (*P* < .001), 30-day reoperation rates (*P* < .001), routine discharge rates (*P* < .001), length of stay (*P* < .001), and rates of all surgical and medical complications assessed with the exception of urinary tract infection (*P* < .001). Specifically, “high-risk” patients demonstrated increased risks of superficial surgical site infection (*P* = .026), deep surgical site infection (*P* < .001), and wound dehiscence (*P* = .025; Table III).

**Table III:** Comparisons of complication rates between high-risk and low-risk clusters.

Variables	Low risk (N = 4468)	High risk (N = 202)	*P*
Pulmonary Embolism	12 (0.3%)	5 (2.5%)	**<.001**
CVA/Stroke with neurological deficit	0 (0.0%)	6 (3.0%)	**<.001**
Complication from DVT/thrombophlebitis	0 (0.0%)	18 (8.9%)	**<.001**
Urinary Tract Infection	36 (0.8%)	4 (2.0%)	.167
Pneumonia	19 (0.4%)	14 (6.9%)	**<.001**
Sepsis	5 (0.1%)	13 (6.4%)	**<.001**
Unplanned Intubation	7 (0.2%)	10 (5.0%)	**<.001**
Progressive Renal Insufficiency	0 (0.0%)	8 (4.0%)	**<.001**
On ventilator > 48 hours	0 (0.0%)	7 (3.5%)	**<.001**
Complication from cardiac arrest requiring CPR	0 (0.0%)	7 (3.5%)	**<.001**
Complication from Myocardial infarction	0 (0.0%)	5 (2.5%)	**<.001**
Bleeding transfusions intraoperative or postoperative	236 (5.3%)	41 (20.3%)	**<.001**
Complication from acute renal failure	0 (0.0%)	2 (1.0%)	**<.001**
Wound Complication	2 (0.1%)	11 (5.4%)	**<.001**
Superficial incisional SSI	13 (0.3%)	3 (1.5%)	**.026**
Organ space SSI	0 (0.0%)	8 (4.0%)	**<.001**
Would disruption	0 (0.0%)	1 (0.5%)	**.025**
Unplanned reoperation	21 (0.5%)	83 (41.1%)	**<.001**
Readmission	88 (2.0%)	90 (44.6%)	**<.001**
Death	16 (0.4%)	18 (8.9%)	**<.001**
Length of total hospital stay	11.6 (12.8)	18.5 (15.1)	**<.001**
Nonroutine discharge	553 (12.4%)	84 (41.6%)	**<.001**
Reoperation diagnosis			**<.001**
Fracture nonunion	0.0%	17 (8.4%)	
Hematoma	0.0%	6 (3.0%)	
Infection	0.0%	13 (6.4%)	
Instability	0.0%	1 (0.5%)	
Mechanical complication	0.0%	21 (10.4%)	
None	4468 (100.0%)	144 (71.3%)	
Reoperation surgery			**<.001**
Revision ORIF	0.0%	22 (10.9%)	
Closed reduction	0.0%	1 (0.5%)	
Explant	0.0%	10 (5.0%)	
Irrigation and Debridement	0.0%	34 (16.8%)	
Miscellaneous	0.0%	4 (2.0%)	
None	4468 (100.0%)	131 (64.9%)	
Related readmission diagnosis			**<.001**
Fracture nonunion	0 (0.0%)	12 (5.9%)	
Cardiac	4 (0.1%)	0 (0.0%)	
GI	5 (0.1%)	6 (3.0%)	
Heme	0 (0.0%)	4 (2.0%)	
Pulmonary	0 (0.0%)	3 (1.5%)	
Renal	0 (0.0%)	6 (3.0%)	
Hematoma	0 (0.0%)	5 (2.5%)	
Infection	0 (0.0%)	4 (2.0%)	
Mechanical complication	0 (0.0%)	11 (5.4%)	
Miscellaneous	0 (0.0%)	2 (1.0%)	
None	4459 (99.8%)	149 (73.8%)	
Unrelated readmission diagnosis			**<.001**
Cardiac	4 (0.1%)	0 (0.0%)	
GI	0 (0.0%)	1 (0.5%)	
Pulmonary	0 (0.0%)	1 (0.5%)	
Renal	0 (0.0%)	2 (1.0%)	
Infection	0 (0.0%)	2 (1.0%)	
None	4464 (99.9%)	196 (97.0%)	

*CVA*, cerebrovascular accident; *DVT*, deep venous thrombosis; *CPR*, cardiopulmonary resuscitation; *SSI*, surgical site infection; *ORIF*, open reduction internal fixation; *GI*, gastrointestinal; *Heme*, hematology.

Categorical variables reported as counts (%).

Bold values represent statistical significance (*P* < .05).

#### Supervised machine learning models

The stepwise logistic regression fitted using the same set of predicted features achieved an AUROC of 0.756, with a calibration intercept of 0.003 (95% confidence interval [CI]: −0.005 to 0.007) and a calibration slope of 0.096 (0.951-1.041). The strongest predictors of high-risk cluster membership included ASA classification 3 (odds ratio [OR]: 5.142, 95% CI: 1.25-6.55, *P* = .023) or 4 (OR: 7.692, 95% CI: 1.7-9.2, *P* = .008) and history of wound infection/open wound (OR: 2.318, 95% CI: 1.03-3.13, *P* = .043) (Table IV).

**Table IV:** Multivariable logistic regression for variables associated with high-risk patients.

Variables	OR	95% CI	*P*
Current smoker within 1 yr	1.53	(1.06-1.9)	**.023**
Open wound	2.32	(1.03-3.13)	**.043**
ASA Classification 3 (severe)	5.14	(1.25-6.55)	**.023**
ASA Classification 4 (life-threatening)	7.69	(1.7-9.21)	**.008**
Total operation time in min	1.00	(1-1.01)	**<.001**

*OR*, odds ratio; *CI*, confidence interval; *ASA*, American Society of Anesthesiologists.

Bold values represent statistical significance (*P* < .05).

**Figure 1:**
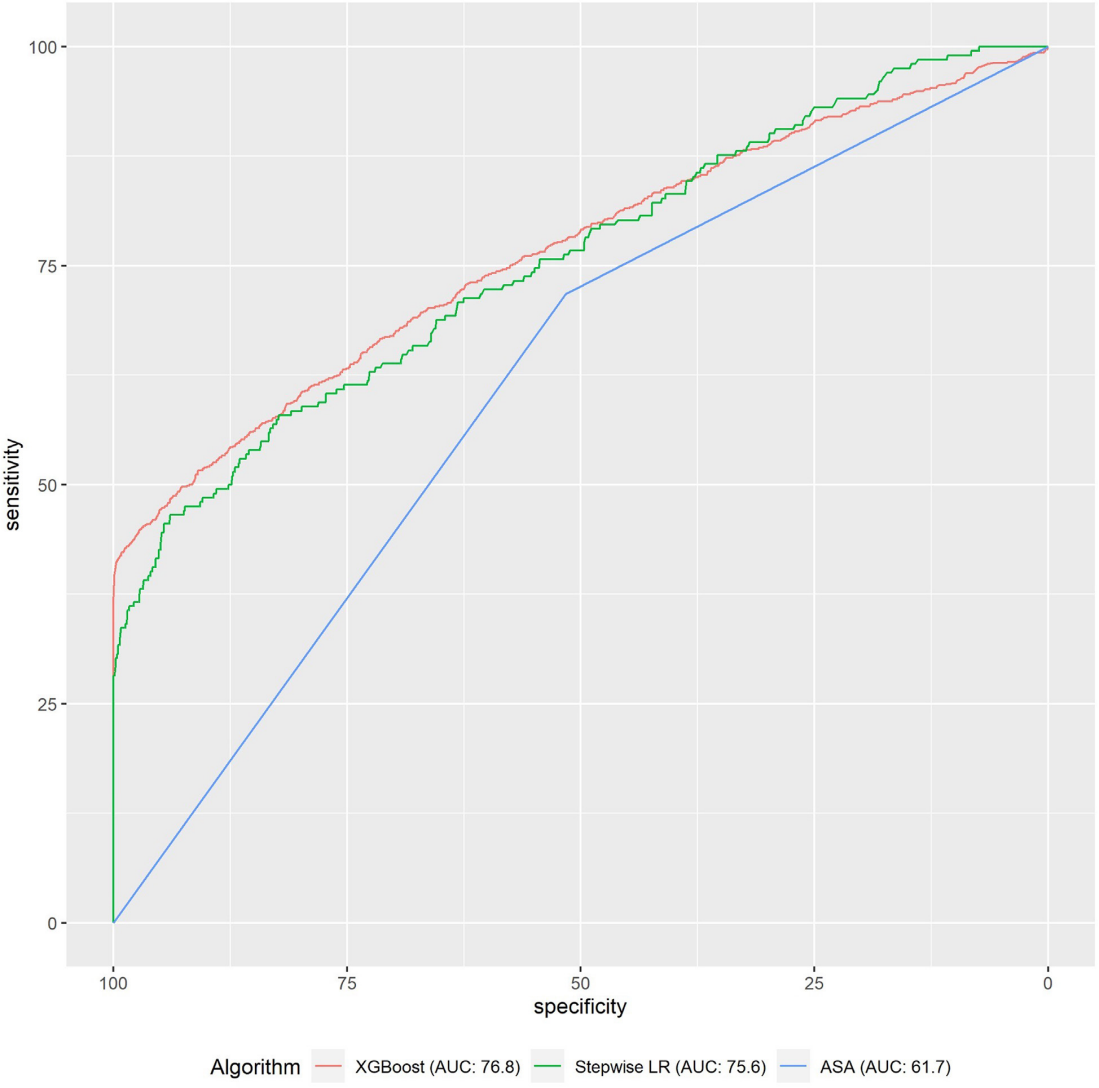
Area under the curve for receiver operating curves for logistic regression model, XGBoost, and for ASA classification algorithms.

The XGBoost model demonstrated optimal performance compared to logistic regression by achieving an AUROC of 0.768, with a calibration intercept of 0.005 (95% CI: 0-0.01) and calibration slope of 0.995 (95% CI: 0.991-0.998). The ASA classification demonstrated an AUROC of 0.617. Calibration intercept and calibration slope were unable to be calculated for ASA since its output is binary and does not provide predicted probabilities. With the ASA model, patients are treated equally regardless of their level of risk. A comparison of AUROC is provided in Figure 1.

**Figure 2:**
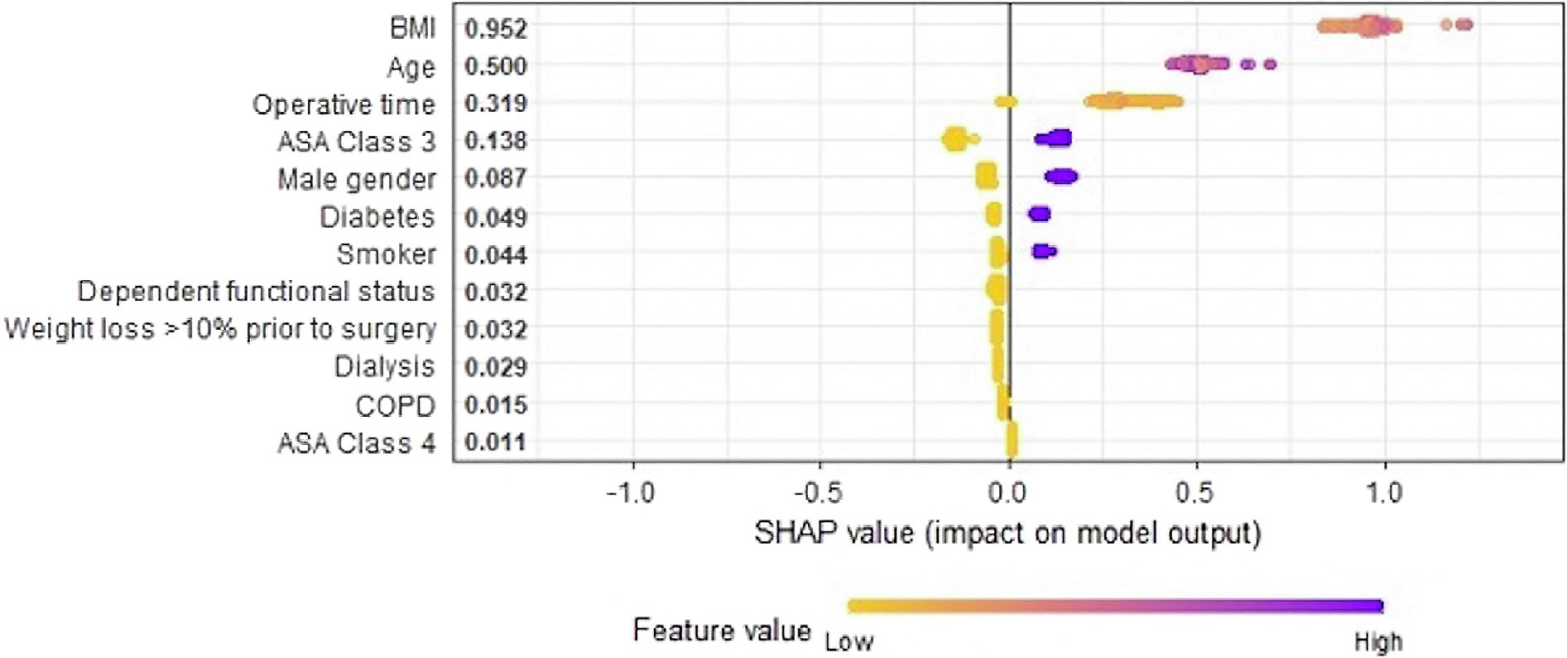
Summary plot of Shapley values produced using a gradient-boosting model. Specifically, the global Shapley values are plotted on the X-axis with variable contributions on the Y-axis, numbers next to each input name indicate the mean global Shapley value, and gradient color indicates feature value. For example: patients with a greater BMI (indicated by *purple dots*) are assigned a higher predicted probability of being in the high-risk cluster (positive SHAP value on the X-axis). Each point represents a row in the original dataset. Dichotomized categorical variables included ASA class (3 and 4), gender, diabetes, smoker, functional status (dependent vs. independent), weight loss, dialysis, and COPD.

Shapley additive explanations were plotted to demonstrate the contributions of input features to patient membership in the high-risk cluster. The strongest contributions to high-risk cluster membership of a new patient included the following: increased body mass index (BMI), increased age, increased operative time, ASA class 3 or 4, male gender, smoking history, and diabetes (Fig. 2).

## Discussion

In this investigation, the authors used the ACS-NSQIP database to develop ML algorithms that identified “high-risk” patients for 30-day complications, readmissions, reoperations, nonroutine discharge, and prolonged length of stay following ORIF of proximal humerus fractures. As healthcare continues its transition toward bundled payment models where the fiscal responsibility of complications within the 90-day global period falls upon physicians and healthcare systems, it is becoming more important for surgeons to optimize preoperative risk. ML may offer an automated method to assist with this process especially when tailored to specific diagnoses. Although this model is not comprehensive, it provides a tool that allows clinicians to make informed decisions regarding the risk of short-term complications following ORIF for proximal humerus fractures.

ORIF of proximal humerus fracture is commonly performed for displaced fractures or fracture dislocations. Early outcomes were fraught with high rate of complications.^3^^,^^7^^,^^19^^,^^21^^,^^52^^,^^55^^,^^62^ As a result, several improvements were made which included the use of endosteal implants (ie, fibular strut allograft), tension band suturing of the rotator cuff, valgus positioning of the humeral head, adequate plate placement, and use of calcar screws, and selective use of endosteal allograft.^5^^,^^23^^,^^54^^,^^59^ Despite these improvements, one study still reported an overall failure rate of 44%, with many failures occurring within 6 months of ORIF.^4^ However, it is important to note that this does not account for postoperative medical complications, such as deep vein thrombosis, cardiac arrest, or transfusion. In patients undergoing reverse total shoulder arthroplasty, an underlying diagnosis of proximal humerus fractures is associated with a higher rate of postoperative complications.^31^ Therefore, it is imperative that physicians and healthcare systems understand the complication risk of these injuries to appropriately allocate resources to optimize outcomes and improve efficiency of healthcare expenditure. Yi et al demonstrated that the ASA classification, modified frailty index, and modified Charlson Comorbidity Index had a limited ability to predict complications following surgical management of proximal humerus fractures. However, these metrics had some utility as a screening tool due to its high negative predictive value.^63^ Therefore, predictive models should be developed and used to identify patients with high risk of complications following ORIF of proximal humerus fractures.

ML algorithms are increasingly being developed and used for improved preoperative risk assessment within orthopedic surgery.^12^ Increased comorbidities coincide with increased resource utilization in patients undergoing operative and nonoperative management of proximal humerus fractures.^32^ The acuity of fractures results in a reduced health state and does not afford patients the opportunity to enhance their health prior to operative intervention. As a result, patients are susceptible to inferior outcomes and complications secondary to a poorer symptom state. This is important because bundled payment models are becoming commonplace in healthcare, and they must account for additional costs associated with the episode of care. Bundled payment models are incompatible with the acuity and complexity of fracture and place the financial burden of complications and readmissions upon physicians. The use of ML for preoperative risk assessment may allow for a tiered calculation of cost and allow for informed decision-making. Supervised ML has seen significant utilization in prediction modeling in orthopedics;^12^ however, these models are restricted to single-outcome prediction, and the number of models required for comprehensive patient-specific risk prediction are cumbersome. The present study combines unsupervised and supervised ML, using the former to elucidate meaningful risk subgroups and then the latter for prediction of subgroup membership of new patients. This novel approach provides an opportunity to counsel patients on a multitude of postoperative complications with the use of a single model. Moreover, instead of a human-designated stratifying variable, the risk clusters are generated based on intrinsic structure within the patient cohort. Therefore, the risk factors that are identified are less arbitrary. In other words, clusters are developed by the model to identify predictors of high risk (ie, BMI and age) instead of the researchers arbitrarily stratifying the patient cohort based upon a variable of interest. This provides a data-driven approach to identify high-risk patients, instead of a subjective method.

Several factors for high-risk patients were identified, including open fracture, tobacco use, higher ASA classification, and operative time. The use of tobacco has been reported as a risk factor for nonunion following ORIF for proximal humerus fractures^49^ and is a risk factor for surgical site complications, readmissions, complications, and reoperations after elective upper extremity surgery.^17^ However, its impact on immediate postoperative complications following ORIF of proximal humerus fractures is yet to be clearly elucidated. Furthermore, operative time has been identified as a risk factor for complications following several orthopedic procedures, including total joint arthroplasty,^9^ rotator cuff repair,^2^^,^^8^ ORIF of ankle fractures,^22^ anterior cervical discectomy and fusion,^39^ and anterior cruciate ligament reconstruction.^1^ Longer operative time may be a risk factor for complications; however, it may also be a byproduct for complexity of the fracture. Despite subjectivity and concerns regarding inter-rater reliability,^47^^,^^50^^,^^57^ the results of the present investigation highlight the utility of the ASA classification in identifying high-risk patients as ASA class 3 and 4 were critical variables in the ML algorithms. However, the area under the curve for ML algorithms was greater than that of ASA classification. This suggests the utility of ML algorithms in predicting outcomes as it can adapt its output to individualized patients.

Much has been discussed regarding the surgical treatment options in patients with proximal humerus fractures. Especially when considering that ORIF has been shown to produce superior functional outcomes, while reverse total shoulder arthroplasty is associated with fewer complications and revisions.^29^^,^^34^ More recent analysis suggests that reverse total shoulder arthroplasty results in greater range of motion, increased complications, but fewer revisions than ORIF.^56^ Nonetheless, ORIF is typically favored in younger, medically fit patients with displaced fractures or with fracture dislocations,^48^ while reverse total shoulder arthroplasty is performed in elderly patients.^20^ Development of a patient-specific treatment algorithm can result in reliable return to preoperative quality of life with reduced complication and revision rates.^51^ The results of this investigation will enable clinicians to identify patients who are at “high risk” for complications and readmissions and help tailor their treatment modality to fit the patient’s risk profile.

### Limitations

It is imperative that the results of this investigation be interpreted within the confines of its limitations. ML research is exponentially increasing in orthopedics. Despite the utility of these models, there are inherent limitations. It is limited by the quality of the data which were used in development of the model. Any miscoding or noncoding in the database decreases the quality of the model. However, the ACS-NSQIP database has several quality assurances programs, such as random internal audits performed biweekly, to ensure the accuracy and quality of the data.^16^^,^^58^ ML algorithms have significant utility; however, a gap exists between their statistical prowess and clinical utility as the results may be difficult to interpret by many clinicians. It is important that clinicians do not interpret every result as clinically significant. This is akin to the concept that statistically significant results do not equate to clinical significance. Furthermore, these models may not be plausible to implement in clinical practice due to the technology, infrastructure, cost, and necessity for updates to maintain accuracy as indications for treatment evolve.^40^ The analysis is limited by variables that are recorded within the ACS-NSQIP database. Therefore, additional variables, such as number of fracture fragments, dislocation, concomitant injuries, indication for surgery, type of fixation, surgical approach, postoperative rehabilitation protocol, weight-bearing limitations, use of tranexamic acid, and patient medications are not included in this analysis. These variables can have a significant impact upon postoperative complications. Furthermore, complications are limited to within 30 days following surgery. Many procedure-specific complications such as nonunions, malunions, and postoperative dislocations are not present within the database and will usually not occur within the 30-day window. Additionally, this investigation is retrospective, which does not allow for control of baseline demographics. The results of this investigation are also subject to selection bias as this database is comprised of large healthcare facilities. Smaller hospitals or surgery centers are unlikely to be represented in this analysis. Thus, the external validity of this investigation may be limited.

## Conclusion

Unsupervised ML identified that “high-risk” patients have a higher risk of complications (8.9%) than “low-risk” groups (0.4%) with respect to 30-day complication rate. A supervised ML model selected greater BMI, increasing age, ASA class 3, increased operative time, male gender, diabetes, and smoking history to effectively predict “high-risk” patients.

## Disclaimers:

Funding: No funding was disclosed by the authors.

Conflicts of interest: The authors, their immediate families, and any research foundation with which they are affiliated have not received any financial payments or other benefits from any commercial entity related to the subject of this article.
