# Supplementary material for: Identifying clinically meaningful subgroups following open reduction and internal fixation for proximal humerus fractures: a risk stratification analysis for mortality and 30-day complications using machine learning
Source: JSES Int. 2024 May 6;8(5):932–40. doi: 10.1016/j.jseint.2024.04.015 (PMC11401551; doi:10.1016/j.jseint.2024.04.015)
Supplement: Supplementary Appendix S1 [file mmc1.docx]

**Appendix 1***:* Internal Validation of Ideal Cluster Number Following K-Means Clustering. Optimal cluster number was determined by minimization of connectivity, AD, and FOM, and maximization of Silhouette.

| Cluster Number | 2 | 3 | 4 | 5 | 6 |
| --- | --- | --- | --- | --- | --- |
| Connectivity | 180.83 | 250.06 | 323.45 | 325.84 | 325.98 |
| Silhouette | 0.05 | 0.03 | 0.03 | 0.03 | 0.02 |
| AD | 88.8 | 92.7 | 96.6 | 111.5 | 132.8 |
| FOM | 2.50 | 2.50 | 2.51 | 2.53 | 2.53 |

*AD*, Average Distance; *FOM*, Figure of Merit
